# Upregulation of HLA‐E by dengue and not Zika viruses

**DOI:** 10.1002/cti2.1039

**Published:** 2018-09-25

**Authors:** Elena Drews, Awadalkareem Adam, Phone Htoo, Elizabeth Townsley, Anuja Mathew

**Affiliations:** ^1^ Division of Infectious Diseases and Immunology University of Massachusetts Medical School Worcester MA USA; ^2^ Department of Cell and Molecular Biology Institute for Immunology and Informatics University of Rhode Island Providence RI USA

**Keywords:** dengue fever, HLA‐E, natural killer, NKG2a

## Abstract

**Introduction:**

The most severe form of dengue virus (DENV) illness, dengue haemorrhagic fever, is characterised by plasma leakage and increased vascular permeability.

**Objectives:**

Given the critical role that endothelial cells play in the pathogenesis of DENV, we wanted to determine whether infection with DENV altered the expression of MHC class I related genes including HLA‐E.

**Results:**

In this study, we provide evidence that HLA‐E but not MICA/B or HLA‐G is upregulated by all four serotypes of DENV in an endothelial cell line human microvascular endothelial cells (HMEC)‐1. In contrast, Zika virus (ZIKV), a related flavivirus, where plasma leakage is not a major manifestation of disease, did not upregulate HLA‐E. We found modest levels of soluble HLA‐E in supernatants from DENV but not ZIKV‐infected cells. Coculture experiments found minimal activation of natural killer (NK) cells in the presence of both uninfected and infected HMEC‐1 cells. HLA‐E induced by DENV infection could not dampen the degranulation of activated NK cells by interacting with its ligand NKG2a.

**Conclusions:**

Our results suggest that while DENV infection induces HLA‐E, the high MHC class I expression on uninfected and infected HMEC‐1 cells may dominate the diverse signals generated between inhibitory and activating receptors on NK cells and ligands on target cells.

## Introduction

Endothelial cells (ECs) are an essential component of the endothelial barrier and play a role in homoeostasis, cell trafficking from the blood to tissues and initiation of both inflammation and the immune response. A hallmark of severe dengue virus (DENV)‐induced disease, dengue haemorrhagic fever (DHF), is vascular leakage.^1^ The transient nature of plasma leakage during DHF^2^ and the absence of strong evidence to indicate direct infection of ECs with DENV *in vivo* in human autopsy studies^3^ suggest that dengue viral products and immune activators elicited by DENV infection in many cells of the innate and adaptive immune system impact EC function.^1^ *In vitro*, DENV infects many kinds of ECs and upregulates VCAM and ICAM on the cell surface. A number of cytokines such as IL‐6, IL‐8 and IFNs are elicited in response to infection.^4^ A focus of many dengue researchers is to understand altered endothelial function by immune activators and DENV products and identify important contributors to the plasma leakage seen in DHF.^5^, ^6^

Endothelial cells are not considered professional antigen‐presenting cells since they express a limited number of costimulatory molecules; however, they express MHC class I and II and can present antigens to T cells.^7^ In addition to classical MHC molecules, ECs also express class I related genes MHC class I polypeptide‐related sequence A (MICA), the nonclassical MHC‐Ib molecule, HLA‐E and the endothelial protein C receptor (EPCR).^7^ These molecules have been recently shown to be important for the regulation and control of infectious agents but whether they provide the endothelium selective functions in response to infection with DENV is unknown.^7^ MICA proteins are constitutively expressed on the surface of ECs and antigen‐presenting cells.^7^ There is genetic evidence to suggest that MICB is a susceptibility locus for severe dengue.^8^, ^9^ Soluble MICB levels were found to be elevated in patients with acute DENV infections.^10^ HLA‐E belongs to the nonclassical group of MHC‐Ib molecules, which include HLA‐E, HLA‐F and HLA‐G in humans, and has been mapped between the HLA‐C and HLA‐A loci on the short arm of chromosome 6.^11^ HLA‐E binds peptides predominantly from signal sequences of HLA‐A, ‐B, ‐C and ‐G, and the surface expression of HLA‐E is influenced by the pattern of HLA expressed in the cell that provides signal peptides. HLA‐E complexed with peptides from some viral infections (cytomegalovirus, Epstein–Barr virus) and bacterial infections (Mycobacterium tuberculosis and Salmonella typhimurium) can interact with T‐cell receptors (TCRs) expressed on CD8 T cells to trigger conventional CTL function.^12^

Natural killer (NK) cells are regulated by many activating and inhibitory receptors including two major families, killer‐like immunoglobulin receptors (KIRs) and C‐type lectin family of receptors CD94 that interact with NKG2.^13^ KIRs interact with classical MHC molecules, while the inhibitory receptor CD94/NKG2a and the activating receptor CD94/NKG2c, present on NK cells and a subset of T cells, interact with HLA‐E.^14^ HLA‐E was recently shown to be upregulated by Japanese encephalitis virus (JEV) infection in human brain microvascular ECs, the endothelial‐like cell line, ECV 304 and human foreskin fibroblasts.^15^, ^16^ Antibodies to TNF and IFNR were able to inhibit the secretion of soluble HLA‐E (sHLA‐E) indicating a role for soluble factors/cytokines in the shedding process. The soluble HLA‐E released from infected cells inhibited IL‐2 and PMA‐mediated ERK 1/2 phosphorylation in two NK cell lines suggesting that HLA‐E could be involved in viral evasion of NK cell responses.^16^

## Results and discussion

### Upregulation of HLA class I and HLA‐E molecules on DENV‐infected HMEC‐1 cells

**Figure 1:**
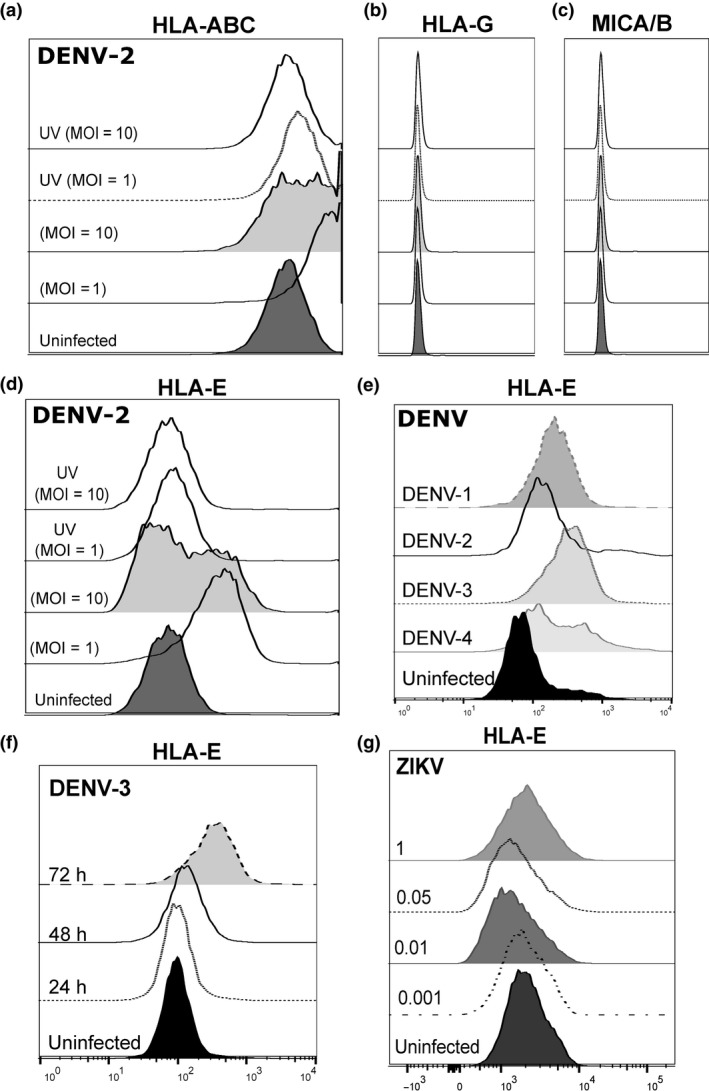
Upregulation of HLA‐E following DENV infection of HMEC‐1 cells. HMEC‐1 cells were infected with live or UV‐inactivated DENV‐2 NGC at the indicated MOIs and stained for the cell surface expression of **(a)** MHC‐1, **(b)** HLA‐G, **(c)** MICA‐/B and **(d)** HLA‐E. HLA‐E expression with **(e)** different serotypes of DENV (MOI = 1), **(f)** different times postinfection with DENV‐3 and with **(g)** ZIKV at the indicated MOIs. Analysis was performed 48 h postinfection. Data shown are representative of one of five experiments performed. DENV, dengue virus; HMEC‐1, human microvascular endothelial cells‐1; ZIKV, Zika virus.

We evaluated an endothelial cell line of dermal origin, human microvascular endothelial cells (HMEC)‐1, for the ability to support DENV infection. We infected HMEC‐1 cells with a laboratory strain of DENV‐2 (MOI = 1 and 10) and assessed the infectivity and the expression of classical MHC molecules and related class I molecules on the cell surface using flow cytometry. Expression of MHC class I molecules on HMEC‐1 cells was high, and it was further upregulated after DENV infection as has been reported previously^17^ using the pan HLA class I specific mAb W6/32 (Figure 1a). We found no expression of HLA‐G and MICA/B on HMEC‐1 cells (Figure 1b, c) but in contrast, HLA‐E was significantly upregulated (Figure 1d) following infection with DENV‐2. UV‐inactivated DENV‐2 did not change the expression of HLA‐E suggesting that active replication was needed for its induction. All four serotypes of DENV upregulated HLA‐E on HMEC‐1 cells (Figure 1e). Peak expression was detected 48–72 h postinfection (Figure 1f) with some variation among serotypes and strains of DENV (data not shown). In contrast, Zika virus (ZIKV), a related flavivirus that does not induce significant plasma leakage, did not upregulate HLA‐E (Figure 1g).

**Figure 2:**
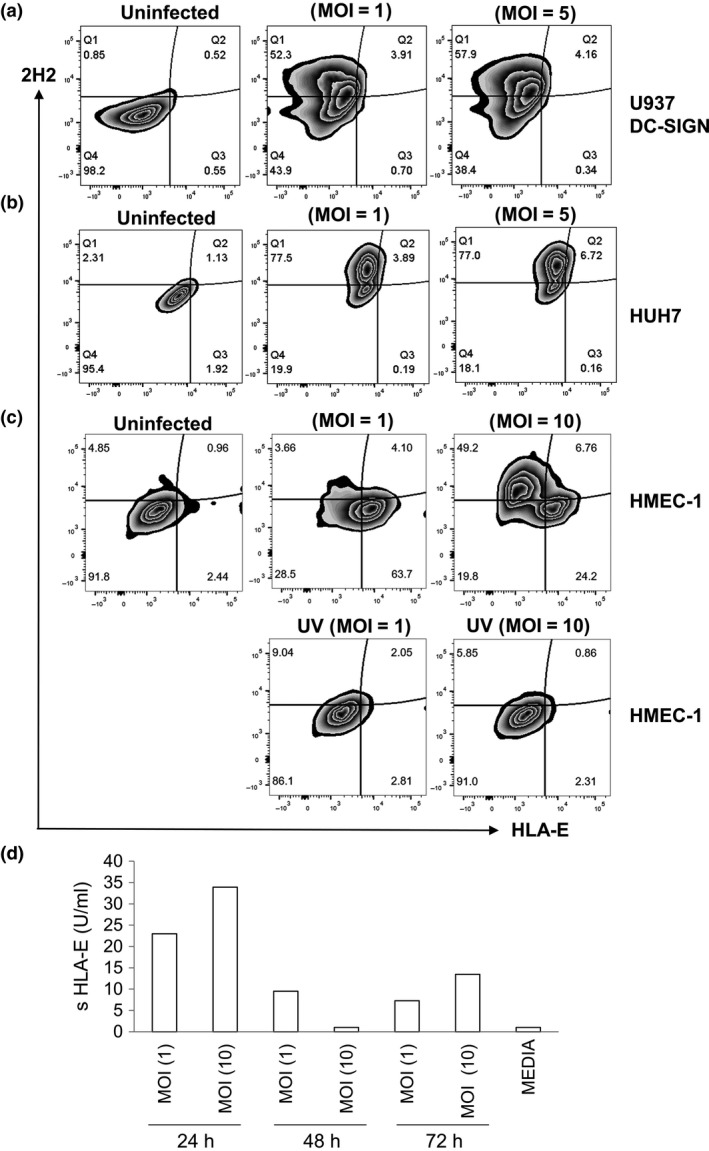
Upregulation of HLA‐E on susceptible cell lines. Flow cytometric analysis of **(a)** U937 DC‐SIGN **(b)** Huh7 and **(c)** HMEC‐1 cells infected with DENV at the indicated MOIs, 48 h postinfection. Cells were stained for HLA‐E, permeabilised and assessed for DENV infection using a labelled antibody 2H2 directed against the prM protein of DENV. Data are representative of one of three experiments performed. **(d)** ELISA for sHLA‐E using cell culture supernatants from DENV‐2‐infected HMEC‐1 cells at the indicated time points and MOIs. DENV, dengue virus; HMEC‐1, human microvascular endothelial cells‐1.

We were interested to know whether HLA‐E was expressed on DENV‐infected cells or bystander HMEC‐1 cells. We first infected two susceptible cell lines with DENV‐U937 expressing DC‐SIGN, and Huh 7. As expected, we found significant infection of these cells by flow cytometry using the well‐characterised monoclonal antibody 2H2 which recognises the premembrane (prM) protein of DENV.^17^, ^18^ However, while we found significant infection in Huh7 and U937 DC‐SIGN cells (50–70% 2H2+ cells), there was no upregulation of HLA‐E 48 h postinfection (Figure 2a, b). In contrast, HMEC‐1 cells infected with DENV (MOI = 1) were poorly infected (approximately 3.66% 2H2+ cells), while over 60% cells upregulated HLA‐E, 48 h postinfection. At a higher MOI (MOI = 10), we were easily able to detect 2H2+ HMEC‐1 cells but most of the HLA‐E upregulation was detected on bystander cells and not virally infected (2H2+) cells (Figure 2c). UV‐inactivated DENV neither increased viral infection nor upregulated HLA‐E in HMEC‐1 cells. The present observations suggest that DENV but not ZIKV upregulates the nonclassical MHC molecule HLA‐E but not HLA‐G, MICA or B in HMEC‐1 cells. As HLA‐E is dependent on signal sequences from HLA class I molecules for its expression,^19^ it is possible that DENV‐induced expression of HLA‐E is mediated through an indirect mechanism since class I is also upregulated in these cells. Our data also support soluble factors induced by DENV infection being responsible for the increased expression of HLA‐E on bystander HMEC‐1 cells as has been shown recently for JEV.^15^

### DENV induces moderate levels of soluble HLA‐E

The functional significance of sHLA‐E molecules is still poorly understood especially in the context of viral evasion of immune responses.^20^ Low levels of sHLA‐E have been detected in the plasma of patients with chronic HBV.^21^ HLA‐E released from JEV‐infected cells blocked IL‐2‐mediated phosphorylation of ERK1/2 in the human NK cell lines suggesting that sHLA‐E could play a role in the regulation of immune responses.^16^

We infected HMEC cells with all four serotypes of DENV and collected supernatants at different times point postinfection. Supernatants from HMEC‐1 cells infected with DENV‐2 had low levels of sHLA‐E compared to supernatants from uninfected HMEC‐1 cells with peak expression detected at 24 h (Figure 2d). Supernatants from cells infected with the other three DENV serotypes and ZIKV also had low levels of sHLA‐E (data not shown). In contrast to JEV infection,^15^, ^16^ we found robust expression of HLA‐E on the cell surface and minimal to moderate levels of sHLA‐E in supernatants of infected HMEC‐1 cells.

### HLA‐E expressed on HMEC‐1 cells following DENV infection does not modulate NK cell function

**Figure 3:**
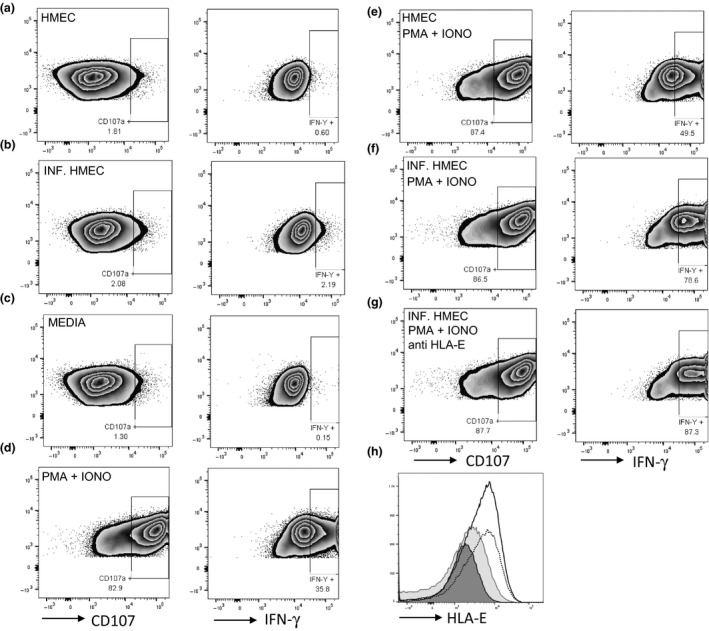
Degranulation and cytokine production by NK92 cells. **(a and b)** NK92 cells were unstimulated or stimulated with PMA + Iono. NK92 cells were added to HMEC‐1 cells that were **(c and e)** uninfected or **(d, f and g)** infected approximately 30 h prior with DENV‐4 (MOI = 10). CD107a degranulation and IFN‐γ expression in NK92 cells were assessed following no stimulation or stimulation with PMA + Iono for approximately 18 h in the presence of Golgi Stop and Plug. **(g)** Anti‐HLA‐E antibody was added at 5 μg mL^−1^ 24 h postinfection. Data are representative of one of two experiments performed. **(h)** Histogram of HLA‐E expression on uninfected (filled dark grey histogram), infected (open solid histogram), infected cells treated with anti‐HLA‐E (filled light grey histogram) or control antibody (open dotted histogram). DENV, dengue virus; HMEC‐1, human microvascular endothelial cells‐1; NK, natural killer.

To determine whether there was a functional consequence of HLA‐E upregulation on the surface of HMEC‐1 cells following DENV infection, we measured the degranulation of NK cells using the marker CD107a and IFN‐γ intracellular expression in coculture experiments. We used an NK cell line, NK92 known to express NKG2a, the well‐recognised inhibitory ligand of HLA‐E.^14^ We added NK92 cells in media to HMEC‐1 cells that were either uninfected or infected 24 h prior with DENV. We found minimal degranulation of CD107a and IFN‐γ expression in NK92 cells that were incubated with either uninfected or DENV‐infected HMEC cells (Figure 3a, b) which is in contrast to findings by Siren *et al*. 22 who reported influenza and Sendai virus‐infected macrophages induced IFN‐γ gene expression and protein production in NK92 cells. We speculate that the high expression of class I (known to dampen NK cell responses) on HMEC‐1 cells, which was further upregulated by DENV infection, may have provided a strong inhibitory signal to NK92 cells thus preventing their activation.

Since NK cells are known to be activated in PBMC from patients undergoing acute dengue infections,^23^, ^24^ we stimulated NK92 cells with PMA + Ionomycin known to upregulate CD107 and IFN‐γ expression (Figure 3c, d). We asked whether HLA‐E induced by DENV infection in HMEC‐1 cells could attenuate IFN‐γ production and the degranulation of activated NK92 cells in our coculture system. Unexpectedly, we found no difference in CD107 degranulation in activated NK92 cells added to infected or uninfected HMEC cells (Figure 3e, f). Instead, IFN‐γ expression was increased in the presence of infected HMEC cells. To determine whether the increase in IFN‐γ expression was dependent on HLA‐E, we used an antibody to block HLA‐E and we found a similar increase in IFN‐γ expression under these conditions. These results indicate to us that HLA‐E induced by DENV infection in HMEC cells was not responsible for the increased IFN‐γ expression in NK92 cells (Figure 3g).

Using our coculture conditions, the cytolytic activity and cytokine expression in NK92 cells expressing the inhibitory receptor NKG2a was not affected by HLA‐E upregulation on HMEC‐1 cells. NK cells expressing the inhibitory (NKG2a) and activating (NKG2c) ligands for HLA‐E have recently been shown to be upregulated on NK cells during acute DENV infection.^25^ Additional studies are required to determine whether NKG2c+ NK cells are impacted by HLA‐E expression on ECs. Furthermore, how NK cells respond to ligands including HLA‐E following DENV infection of other target cells such as primary dendritic cells and monocytes may differ from responses to ECs and are worthy of future study.

## Methods

### Passage and infection of cell lines

HMEC‐1 cells were grown in MDCB 131 media (ThermoFisher Scientific, Waltham, MA, USA) with 10% FBS, supplemented with 10 ng mL^−1^ epidermal growth factor and 1 μg mL^−1^ hydrocortisone and passaged in T75 flasks. For infections, HMEC‐1 cells were trypsinised with 1:4 diluted trypsin/EDTA and 1.5 × 10^5^ HMEC‐1 cells were plated on 12‐well tissue culture plates. HMEC‐1 cells were infected with different strains of DENV (DENV‐1 Hawaii, DENV‐2 NGC, DENV‐3 CH53489 and DENV‐4 814669) at the indicated multiplicity of infection (MOI). Huh7 cells were grown in DMEM media (Mediatech Inc., Manassas, VA, USA) with 10% FBS. U937 DC‐SIGN cells were grown in RPMI 1640 media with 10% FBS (Sigma‐Aldrich, St Louis, MO, USA). Cells were incubated with DENV in 250 μL for 90 min. After infection, 750 μL of the indicated media was added, and infected HMEC‐1 was incubated at 37°C in 5% CO_2_ for 48 h.

### Staining for class I related molecules and DENV

Uninfected and infected HMEC‐1 and Huh 7 cells were trypsinised and washed in fluorescence activated cell sorting buffer (PBS/2%FBS/0.1% sodium azide). HMEC‐1, U937 DC‐SIGN and Huh 7 cells were stained with an antibody to HLA‐E (clone 3D12), HLA‐G (clone 87G), MICA/B (clone 6D4) for 20 min, washed and permeabilised in 200 μL Cytofix/Cytoperm (BD Biosciences, San Jose, CA, USA) for 20 min at room temperature. Cells were washed in 1× Perm Wash buffer (BD Biosciences) and stained with DL650 labelled DENV‐specific antibody 2H2 for 30 min.

### Soluble HLA‐E ELISA

HMEC‐1 cells were infected with at the indicated MOIs. Culture supernatants were collected after 24, 48 and 72 h, spun at 845 *g* for 10 min, and stored at −80°C. Human leucocyte antigen E (HLA‐E) ELISA was performed on cell culture supernatants according to manufacturer's instructions using an ELISA kit from TSZ ELISA. A serial standard was used to create a standard curve that ranged from 3.12 to 200 U mL^−1^. Each sample was tested in duplicates.

### Degranulation and cytokine assay

NK92 cells (2.5 × 10^5^) grown in specialised media and maintained as described^26^ were added to HMEC‐1 cells grown in 12‐well plates that were either uninfected or infected with DENV approximately 30 h prior. PMA (100 ng mL^−1^) + Ionomycin (50 ng mL^−1^) (PMA + Iono) was added to the indicated wells. CD107 antibody (clone H4A3) was added to wells containing NK92 cells. After 1 h, Golgi Stop and Plug was added. After 18 h, NK92 cells were removed from the plate and stained for CD56 (clone HCD56), NKG2A (clone Z199) and intracellular expression of IFN‐γ (clone B27). In experiments where we blocked HLA‐E expression, 5‐10 μg mL^−1^ of anti‐HLA‐E was added to the indicated wells 24 h postinfection. Data were collected on a BD LSR and analysed using FlowJo version X software (Tree Star Inc, Ashland, OR, USA).
